# Heterogeneity of dormitory interpersonal conflict coping style and its negative emotional characteristics among college students: based on latent profile analysis

**DOI:** 10.3389/fpsyg.2025.1606721

**Published:** 2025-09-09

**Authors:** Junxing Pan, Xiaoyun Zhao, Cui Lyu, Lili Xu, Ying Zhu

**Affiliations:** 1 Department of Student Affairs, Huaibei Normal University, Huaibei, China; 2 School of Education, Huaibei Normal University, Huaibei, China

**Keywords:** dormitory interpersonal relationships, interpersonal conflict coping style, latent profile analysis, college student, negative emotions

## Abstract

**Background:**

Dormitory interpersonal conflict coping style has an important impact on the quality of college students’ dormitory interpersonal relationships and emotional experiences. Still, previous studies have ignored the individual differences among college student groups, making it difficult to effectively classify these groups from the perspective of dormitory conflict coping style. This study aims to explore the potential categories of conflict coping styles in college students’ dormitories and their demographic characteristics, and to analyze the emotional differences among college students in different types of dormitory conflict coping groups, with the expectation of providing theoretical references for improving interpersonal relationships in college students’ dormitories.

**Methods:**

A convenience cluster sampling method was used to select 1,408 college students from a university in Anhui Province, China, to conduct an online survey. The Dormitory Interpersonal Relationship Questionnaire, the Dormitory Interpersonal Conflict Coping Style Questionnaire, and the Depression-Anxiety-Stress Scale Short Chinese Version were used for the investigation. First, we used “individual-centered” latent profile analysis to explore the categories of dormitory interpersonal conflict coping style; second, we used multifactorial binary logistic regression to explore the relationship between demographic variables and the potential categorization of dormitory interpersonal conflict coping style; and lastly, we used a difference-in-differences test to explore the emotional characteristics of different dormitory interpersonal conflict coping style groups.

**Results:**

The dormitory interpersonal conflict coping style group of Chinese college students was divided into two potential categories: the positive coping group (85.01%, 1,197 students) and the negative coping group (14.99%, 211 students). The results of binary logistic regression analysis showed that gender, grade, and type of specialty were all correlated with dormitory interpersonal conflict coping style in college: male students (OR = 1.86), senior students (OR sophomore = 1.44; OR juniors = 1.70), and non-literature and history majors (OR science and engineering = 1.57; OR arts and sports = 2.34) were more likely to choose negative responses to interpersonal conflicts in the dormitory (all *p* < 0.05). The positive coping group scored significantly lower on negative emotions than the negative coping group.

**Conclusion:**

There is significant group heterogeneity in the dormitory interpersonal conflict coping style of Chinese college students. Schools should guide college students to choose appropriate interpersonal conflict resolution strategies to create positive interpersonal relationships in dormitories.

## Introduction

Since 1999, when universities expanded in China, more and more students have been enrolled in higher education. As of 2022, the number of students enrolled in China’s higher education has reached 46.55 million, an increase of nearly 50% in the past decade. Although the size of school buildings is also increasing year by year, the dormitory conditions have not kept up (Punch News, 2024). The vast majority of Chinese university students live in dormitories provided by the university, with a standard of 4–6 people per room, and roommates are randomly assigned (Cao et al., 2024). As the basic living unit of Chinese university campuses, the dormitory is an important place for students to rest, study, and recreate, and dormitory life is an important part of students’ campus life. Dormitory interpersonal relationships are very important interpersonal relationships among Chinese college students during their time at school (Wang, 2008). Dormitory interpersonal relationships refer to the interpersonal connections formed by college students in the process of communication and interaction with dormitory classmates, which is an important social resource for college students during their time at school (Zhao et al., 2019), and has a widespread, important, and direct impact on students’ mental health (Erb et al., 2014). The results have shown that dormitory interpersonal relationships are positively correlated with college students’ life satisfaction, subjective well-being (Yang and Zhang, 2012; Foulkes et al., 2021; Golding et al., 2022), learning attitude, academic performance (Zhang et al., 2012; Pan and Gao, 2017; Hanasono and Nadler, 2012), and general self-efficacy (Lin et al., 2015). It is negatively associated with internalized psychological problems, such as depression and anxiety (Ding et al., 2024; Bhatra et al., 2023), mobile phone addiction (Guo et al., 2021), aggression (Zhao and Su, 2015), etc.

Dormitory members live in the same small space day and night, and interpersonal interactions are frequent, which can easily lead to interpersonal conflicts due to various factors such as individual differences, communication barriers, and different needs (Dong et al., 2012). According to the survey, more than 40% of college students have had conflicts with their roommates (China Youth Net, 2017), and 30%–60% of college students are dissatisfied with their dormitory interpersonal relationships and have varying degrees of conflict with their roommates (Golding et al., 2022; Chen et al., 2012). It can be seen that interpersonal conflict in the dormitory is a common phenomenon. Interpersonal conflict in a dormitory refers to a relationship between dormitory members that is emotionally, verbally, and behaviorally hostile, indifferent, nervous, disharmonious, and even involves fighting with each other due to differences in their needs, interests, goals, or opinions on matters such as study, life, and emotion (Jia, 2009). Studies have shown that the impact and consequences of interpersonal conflict depend on how individuals deal with the conflict rather than the frequency and nature of the conflict itself (Rahim, 1983). Conflict coping styles refer to the behavioral tendencies and patterns that individuals exhibit in the face of interpersonal conflict (Chen et al., 2012). In the face of interpersonal conflicts in dormitories, individuals who adopt appropriate conflict coping styles can eliminate misunderstandings and enhance mutual understanding between the two parties to the conflict, thus improving interpersonal relationships; whereas the adoption of inappropriate conflict coping styles can damage interpersonal relationships and jeopardize the physical and mental health of both parties (Chen et al., 2012). Thomas proposed that the five styles of conflict management are divided into avoiding, competing, accommodating, collaborating, and compromising (Thomas, 1976), and this theory has a wide range of influence. Chinese researcher Jia developed the Dormitory Interpersonal Conflict Handling Questionnaire for Chinese college students with reference to Thomas’s theory and combined it with the interview method, which has four dimensions (cooperation, competition, avoidance, and compliance) (Jia, 2009), and has been more widely used in China. The researchers pointed out that there are four common interpersonal conflict coping styles among Chinese college students: cooperation, competition, avoidance, and compliance (humility), with the highest frequency of use being cooperation, followed by compliance and avoidance, and the lowest being competition. Among them, cooperation is a positive response, competition and avoidance are negative responses, and the nature of compliance is relatively vague (Jia, 2009; Deng et al., 2015; Yang, 2020). The positive coping style refers to an individual’s ability to pay attention to the needs and interests of both parties in conflict during conflict management, face and resolve conflicts with a more proactive attitude, and seek a win-win outcome. Negative coping styles refer to situations where individuals ignore the needs and interests of one or both parties in conflict management, and more often adopt methods such as confrontation, suppression, avoidance, or concession to deal with conflicts, which is not conducive to the effective resolution of conflicts.

Previous studies have shown that there are differences in the impact of different types of interpersonal conflict coping styles on dormitory interpersonal relationships, with cooperation positively correlated with dormitory interpersonal relationships, avoidance and competition negatively correlated with dormitory interpersonal relationships, and compliance either positively correlated or uncorrelated with dormitory interpersonal relationships (Zhao et al., 2019; Yang, 2020). Although the researchers explored the frequency, demographic characteristics, and consequences of different types of dormitory conflict coping styles, there are still the following shortcomings. First of all, previous studies have ignored the individual differences of college students, and it is difficult to effectively categorize college students from the perspective of dormitory conflict response. Second, there are inconsistencies in the results of research on the demographic differences in dormitory conflict coping. In view of the above, this study adopts an individual-centered latent profile analysis method to explore the potential categories and demographic characteristics of conflict coping styles in college students’ dormitories and analyzes the emotional differences among college students in different types of dormitory conflict coping groups, with the aim of providing theoretical references for improving interpersonal relationships in college students’ dormitories.

## Methods

### Participants

From November to December 2024, an online survey of college students from a university in Anhui Province, China, was carried out using the convenient cluster sampling method and relying on the “Wenjuanxing” platform.^1^ A total of 1,408 research subjects were finally included.

Among them, there are 365 boys and 1,043 girls; 816 freshmen, 482 sophomores, and 110 juniors; 657 majors in literature and history, 679 majors in science and engineering, and 72 majors in arts and sports; 903 with home residence in the village or town, 251 with home residence in the county, and 254 with home residence in the city; 255 lived in single-child households and 1,153 lived in non-single-child households; 1,014 people who had residential experience and 394 people did not have residential experience before going to university; 559 people had left-behind experience and 849 had no left-behind experience (“Left-behind experience” refers to the experience that, before the age of 18, at least one parent has been working outside the home for more than half a year and has not been around to take care of them); the average age was (18.94 ± 1.01) years old. All study subjects signed the informed consent form. This study complies with the Declaration of Helsinki.

### Measures

#### General demographic information

Including gender, grade, age, type of major, home residence, whether living in a single-child household, previous accommodation experience, and whether there is any left-behind experience, etc.

#### Dormitory interpersonal relationships

The Chinese version of the Dormitory Interpersonal Relationship Questionnaire was developed by Jiang (2011), with a total of 17 questions, and was scored on a 5-point Likert scale from 1 (not at all) to 5 (completely agreeable). It includes five dimensions: group cognition, verbal communication, sharing behavior, disturbing behavior, and personality characteristics. Some questions are reverse-scored and then summed; the higher the score, the better the dormitory relationship. The Cronbach’s *α* coefficient of the total scale in this study was 0.80.

#### Dormitory interpersonal conflict coping styles

The Chinese version of the Dormitory Interpersonal Conflict Coping Style Questionnaire was developed by Jia (2009). It contains a total of 17 questions, using a 4-point Likert scale from 1 (never used) to 4 (often used). The questionnaire includes four dimensions: competition (4 items), cooperation (5 items), avoidance (4 items), and compliance (4 items). A higher score indicates a higher frequency of use of that coping style. In this study, the Cronbach’s *α* coefficients were 0.75, 0.88, 0.64, and 0.70, respectively. For the compliance dimension, the coefficient was 0.58, but it increased significantly to 0.70 after deleting two items with CITC values lower than 0.40 in this dimension.

#### Negative emotions

The Depression-Anxiety-Stress Scale Short Chinese Version (DASS-21) was revised by Gong et al. (2010). It consists of a total of 21 items, using a 0 (non-conforming) to 3 (always conforming) Likert 4-point scale, including three dimensions: depression, anxiety, and stress, each with 7 items. It measures the depression, anxiety, and stress experienced by an individual in the past week, with higher scores indicating stronger experiences. In this study, the total Cronbach’s α coefficient of the scale was 0.96, and the Cronbach’s α coefficients of depression, stress, and anxiety were 0.90, 0.91, and 0.93, respectively.

### Statistical analysis

Mplus 8.3 software was used to analyze the potential profile of interpersonal conflict coping styles in college dormitories, and the determination of the best model was comprehensively judged by the following indicators: the lower the Akaike Information Criterion (AIC), Bayesian Information Criterion (BIC), and sample size-adjusted aBIC values, the better the model fit; a *p*-values < 0.05 for LMR and BLMR indicates that the current model is significantly better than the model of the previous classification. An Entropy value above 0.80 indicates a high level of classification accuracy, with values closer to 1 indicating greater feasibility (Wang and Bi, 2018). SPSS 25.0 software was used for data analysis. The count data were expressed as frequency and percentage, the *χ*^2^ test was used for comparison between groups, and the influence of each factor on the potential types of conflict coping styles in different dormitories was evaluated by multivariate binary logistic regression analysis, with *p* < 0.05 considered statistically significant.

## Results

### Latent profile analysis of dormitory conflict coping styles in the college

Based on the 1-classification model, the number of profiles was gradually increased to explore the best potential number of categories that could fully explain the relationship between conflict coping style indicators in college dormitories. The results showed that the values of AIC, BIC, and aBIC gradually decreased with the increase in the number of categories, and the lowest values were never found; and the LMR and BLMR indexes were statistically significant in all models (all *p* values < 0.01). Although the Entropy values for categories 3 and 5 are more than 0.90, there are cases where the category probability values are low. In summary, considering the accuracy and conciseness of model selection, two categories were finally selected as the optimal model (see Table 1).

**Table 1:** Fitting information for latent profile analysis of conflict response in college dormitories(*n* = 1,408).

Category	*AIC*	*BIC*	*aBIC*	*Entropy*	*LMR(p)*	*BLRT(p)*	Categorical probability
1	9144.76	9186.76	9161.35	-	-	-	1
2	**8401.33**	**8469.58**	**8428.28**	**0.89**	**<0.01**	**<0.01**	**0.85/ 0.15**
3	7973.04	8067.54	8010.36	0.91	<0.01	<0.01	0.03/ 0.20/ 0.77
4	7820.79	7941.54	7868.48	0.84	<0.01	<0.01	0.19/ 0.68/ 0.09/ 0.03
5	7603.56	7750.56	7661.61	0.90	<0.01	<0.01	0.64/ 0.15/ 0.18/ 0.00/ 0.03
6	7456.31	7629.56	7524.73	0.89	<0.01	<0.01	0.61/ 0.09/ 0.02/ 0.20/ 0.00/ 0.08

The bold values indicate selected results or significant results.

**Figure 1:**
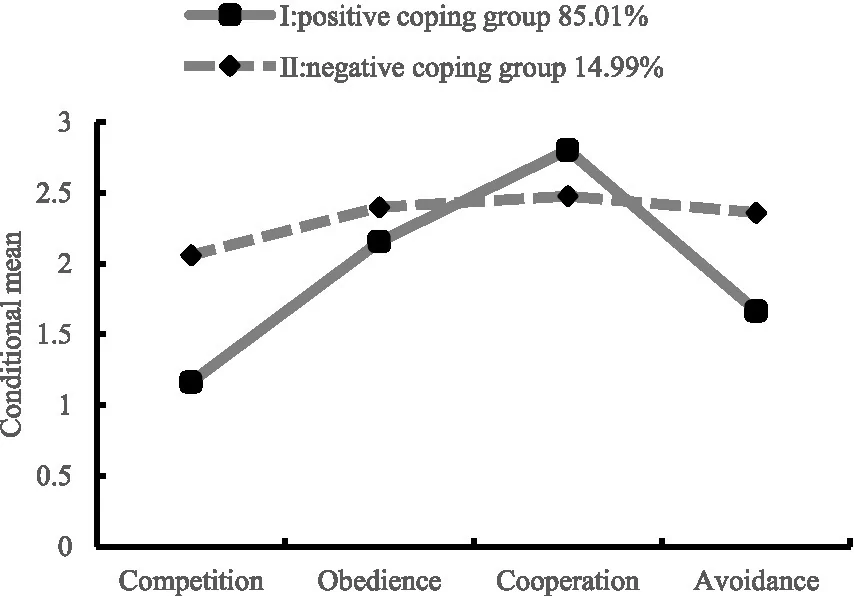
Average scores of potential categories for interpersonal conflict coping style in college dormitories across various dimensions. The horizontal axis represents the dimensions for interpersonal conflicts coping style in college dormitories, and the vertical axis represents the original average scores of the samples in each dimension.

As can be seen from Table 2, there were statistically significant differences in the four dimensions of dormitory conflict coping style and dormitory interpersonal relationship scores between the two potential categories of dormitory conflict coping style (all *p* values < 0.01). The two categories were named according to the scores of the four dimensions of the Dormitory Interpersonal Conflict Coping Style Questionnaire. The scores of the students in category I were significantly lower than those in category II, while the scores of cooperation were significantly higher than those in category II. Therefore, 1,197 students (85.01%) were classified in category I, and 211 (14.99%) were classified in category II as the positive coping group and negative coping group, respectively (see Figure 1).

**Table 2:** Comparison of the scores of different types of college students in dormitory interpersonal relationships, dormitory conflict coping, and their dimensions and emotions (*x* ± *s*).

Potential categories	Number of cases	Dormitory interpersonal relations	Dormitory interpersonal conflict coping style				Negative emotions		
			Competition	Compliance	Cooperation	Avoidance	Depression	Stress	Anxiety
I	1,197(85.01%)	3.98 ± 0.53	1.16 ± 0.21	2.16 ± 0.62	2.80 ± 0.67	1.67 ± 0.44	3.03 ± 3.51	2.58 ± 3.72	2.18 ± 3.23
II	211(14.99%)	3.42 ± 0.53	2.08 ± 0.42	2.40 ± 0.56	2.47 ± 0.58	2.36 ± 0.47	6.64 ± 4.35	6.09 ± 4.52	5.66 ± 4.46
*T*		14.24	−31.08	−5.37	6.89	−21.00	−11.44	−10.65	−10.81
*P*		<0.01	<0.01	<0.01	<0.01	<0.01	<0.01	<0.01	<0.01

### Analysis of influencing factors of conflict coping style in college dormitories

Demographic variables were used as rows, and two potential categories of dormitory conflict coping styles were used as columns. A contingency table analysis was carried out. The results showed that there were statistically significant differences in the distribution of potential categories of dormitory conflict coping styles among college students of different genders, grades, and majors (all *p* < 0.01) (see Table 3).

**Table 3:** The distribution of different groups of college students in the potential categories of dormitory interpersonal conflict coping style.

Group	Options	Number of cases	Dormitory interpersonal conflict coping style		*χ^2^*
			Positive coping group	Negative coping group	
Gender	Male	365	283(77.4)	82(22.5)	21.64**
	Female	1,043	914(87.6)	129(12.4)	
Grade	Freshman	816	707(86.6)	109(13.4)	6.27**
	Sophomore	482	404(83.8)	78(16.2)	
	Junior	110	86(78.2)	22(21.8)	
Type of specialty	Literature and History	657	586(89.2)	71(10.8)	17.69**
	Science and engineering	679	555(81.7)	124(18.3)	
	Arts and Sports	72	56(77.8)	16(22.2)	
Home residence	Village or town	903	766(84.8)	137(15.2)	0.96
	County	251	218(86.9)	33(13.1)	
	City	254	213(83.9)	41(16.1)	
Lived in single-child households	Yes	255	216(84.7)	981(15.3)	0.02
	No	1,153	39(85.1)	172(14.9)	
Pre-university housing experience	Yes	1,014	854(84.2)	160(15.8)	1.79
	No	394	343(87.1)	51(12.9)	
Left-behind experience	Yes	559	465(83.2)	94(16.8)	2.44
	No	849	732(86.2)	117(13.8)	

The figures in parentheses are the composition ratio/%; **P < 0.01.

Multivariate binary logistic regression analysis was performed with two potential categories of dormitory conflict coping style (“positive coping group” as the reference) as the dependent variables, and gender (male students as the reference), grade (freshmen as the reference), and major type (literature and history as the reference) as independent variables. Specifically, male students were 1.86 times more likely to choose a negative coping style than female students; sophomores were 1.44 times more likely to choose a negative coping style; junior students were 1.70 times more likely to choose a negative coping style; science and engineering students were 1.57 times more likely to choose a negative coping style than students majoring in literature and history; and art and sports students were 2.34 times more likely to choose a negative coping style. In other words, female students, first-year students, and students majoring in literature and history are more likely to choose a positive way to deal with interpersonal conflicts in the dormitory (see Table 4).

**Table 4:** Multivariate logistic regression analysis of influencing factors of college students’ dormitory interpersonal conflict coping style [*OR* (95% *CI*)].

Independent variable	Options	Negative coping group	*p*
Gender	Male	1.86(1.35 ~ 2.56)	< 0.01
Grade	Sophomore	1.44(1.04 ~ 2.00)	< 0.05
	Junior	1.70(1.02 ~ 2.83)	< 0.05
Type of specialty	Science and engineering	1.57(1.13 ~ 2.19)	< 0.01
	Arts and Sports	2.34(1.25 ~ 4.39)	< 0.01

Gender in the independent variables is referenced to female students, grade level is referenced to freshman year, and type of major is referenced to arts and history.

### Analysis of the emotional differences of college students in different types of dormitory conflict coping style groups

The independent sample *t*-test was used to compare the differences in the emotional scores of college students in different types of dormitory conflict coping style groups. The results showed that the scores of depression, stress, and anxiety were significantly lower in the positive coping group than in the negative coping group (all *p* < 0.01) (see Table 2).

## Discussion

### Potential categories of dormitory conflict coping style

The results showed that, firstly, college students’ dormitory conflict coping styles could be divided into two potential categories: the positive coping group (85.01%) and the negative coping group (14.99%). It can be seen that there is heterogeneity in college students’ coping with dormitory conflicts, and most students can choose positive coping methods when facing interpersonal conflicts in dormitories, but a small number of groups may lack active and effective interpersonal coping strategies. This is because college students are in a critical period of “self-identity” development; their personalities are gradually improving, and their conflict coping styles are gradually becoming rational and mature, and they can actively choose positive and reasonable coping strategies when facing interpersonal conflicts in the dormitory (Jia, 2009; Deng et al., 2015; Yang, 2020). Secondly, the dormitory interpersonal relationship scores of college students in the positive coping group were higher than those in the negative coping group. This suggests that positive coping styles do help improve the quality of interpersonal relationships in dormitories, while negative coping styles may disrupt interpersonal relationships in dormitories, which is consistent with existing studies (Zhao et al., 2019; Yang, 2020). This reaffirms that the impact and consequences of interpersonal conflict depend on how interpersonal conflict is coped with rather than on interpersonal conflict itself (Rahim, 1983). Finally, the scores of college students in the positive coping group of dormitory conflict in the three dimensions of competition, compliance, and avoidance were lower than those of the negative coping group, while the scores of the cooperation dimension were higher than those of the negative coping group. The coping strategies of the two groups were ranked in the same order, which were cooperation, compliance, avoidance, and competition, which was consistent with the previous research. Numerous studies have shown that the frequency of conflict coping in college dormitories is cooperation, compliance, avoidance, and competition (Jia, 2009; Deng et al., 2015; Yang, 2020). This is in line with the traditional Chinese culture of valuing “harmony,” which reflects the Chinese people’s pursuit of interpersonal harmony (Zhao et al., 2019; Dong et al., 2012; Yang, 2020; Wang, 2022). Cooperation has the nature of prosocial behavior and is a win-win strategy that helps to promote interpersonal harmony (Xiao et al., 2014) and improve interpersonal adaptation (Cen and Nie, 2009), while competitive coping increases interpersonal distress (Zhao and Su, 2015; Fan and Zhang, 2003; Wang, 2017; Sillars, 1980). When cooperative strategies cannot be used to effectively deal with interpersonal conflicts, individuals tend to choose relatively mild adversarial, submissive, and avoidance strategies rather than fiercely adversarial competitive strategies to maintain interpersonal harmony to the greatest extent (Wang, 2022).

### Demographic characteristics of dormitory conflict coping styles

The results showed that gender, grade, and major type were significantly correlated with interpersonal conflict coping styles in college dormitories. Specifically, first, female students are more likely to choose to respond positively to interpersonal conflicts in dormitories, consistent with the results of previous studies (Zhao et al., 2019; Chen et al., 2012; Wang, 2022). Evolutionary psychology research has shown that males are more aggressive than females and are more likely to use negative coping strategies such as competition in the face of interpersonal conflict (Zhao et al., 2019; Fan and Zhang, 2003; Bazezew and Neka, 2017). On the other hand, in the process of individual socialization, adults encourage girls to be gentle and humble, while boys are encouraged to be resolute and brave to compete. This differentiated education is also reflected in the way of coping with interpersonal conflicts, with women more inclined to cooperate and tolerate to seek reconciliation, while men will use competitive aggression more to vent their dissatisfaction with conflict (Deng et al., 2015; Li and Tang, 2010; Wang and Zhao, 2023). Second, first-year students are more likely to choose to respond positively than sophomores and juniors, which is consistent with the results of existing studies (Yang, 2020). The study shows that the cooperation score of freshmen is higher than that of senior students, which may be due to the fact that freshmen have lower interpersonal familiarity. In order to adapt to the unfamiliar environment as soon as possible and improve their sense of belonging, they pay more attention to creating a harmonious dormitory relationship and are more willing to choose a positive way to cope with interpersonal conflicts in the dormitory. As interpersonal familiarity increases, interpersonal relationships in the dormitory become basically stereotyped, and other interpersonal circles outside the dormitory gradually expand. The sources of students’ sense of belonging become diversified, so the willingness of senior students to try to find a win-win solution when facing dormitory conflicts is significantly reduced (Yang, 2020; Wang, 2022). Finally, students majoring in literature and history are more likely to choose to respond positively than students majoring in science and engineering and the arts and sports, which is consistent with the results of previous studies (Yang, 2020; Wang, 2022). Studies have shown that students from the sciences have heavier academic tasks, pay more attention to personal future issues and life values, and their interpersonal autonomy, caring for others, and self-image goals are significantly lower than those of literature and history majors (Yang, 2020). Correspondingly, there are differences in the way they deal with interpersonal conflicts. Arts and sports students may be more sensitive, pay more attention to personal feelings in interpersonal communication and conflict, and have a relatively low willingness to seek a win-win situation.

### Differences in the emotions of college students in different types of dormitory conflict-coping style groups

The results showed that there were significant differences in the emotional scores of college students in the interpersonal conflict coping group across different types of dormitories, and the negative emotions in the positive coping group were significantly lower than those in the negative coping group, which was consistent with the results of previous studies (Zhao et al., 2019). Research shows that there is a two-way relationship between conflict management style and individual emotions. First, individuals with positive emotions are more likely to choose cooperative conflict management. However, individuals with negative emotions are more likely to choose competitive conflict management (Pan, 2009). This can be explained by the theory of emotional contagion. The emotional states of individuals influence each other. The emotions of one party can affect those of the other, triggering similar emotional responses (Hazy and Boyatzis, 2015). In social interaction, individuals’ emotions can influence each other, thereby leading to behavioral synchronization (Herrando and Constantinides, 2021). Therefore, an individual’s emotional state not only affects their own conflict management approach but may also drive the other party to choose a similar conflict management approach. Second, individuals who adopt positive coping styles (e.g., cooperation) tend to have better interpersonal relationships and fewer negative emotions, while individuals who adopt negative coping styles (e.g., competition and avoidance) tend to experience interpersonal distress and more negative emotions (Wang and Zhao, 2023; Liao et al., 2012; Lei et al., 2018). According to Maslow’s hierarchy of needs theory (Baumeister and Leary, 1995), when faced with interpersonal conflicts in the dormitory, which is a common stressful situation, adopting a cooperative coping style can help to increase interpersonal trust, alleviate interpersonal conflicts and stress (Wang, 2017), and satisfy the needs of individual belonging and respect. However, competition and avoidance are not conducive to the resolution of interpersonal conflicts in the dormitory and may even intensify the conflicts, increase interpersonal pressure, and make it difficult to effectively meet the needs of individuals for belonging and respect; and it is easy to experience negative emotions (Wang, 2017; Wang and Zhao, 2023; Liao et al., 2012; Yang et al., 2024). Individuals who adopt a submissive approach tend to accumulate negative emotions by ignoring their own needs in order to satisfy the needs of the other person (Antonioni, 1998).

The conclusion of this study has important implications for the reasonable resolution of interpersonal conflicts in college student dormitories. In the daily management of students and mental health education, it is advisable to promote more about the significant importance of positive interpersonal conflict response methods for resolving conflicts (which are positively correlated with interpersonal harmony and negatively correlated with negative emotions). At the same time, encourage students to reflect on their usual ways of managing interpersonal conflicts, guide them to see and balance the needs and interests of both sides of the conflict, and actively learn and apply positive coping styles (such as cooperation), while using negative coping styles (such as competition and avoidance) less, in order to seek the greatest possible win-win situation.

### Limitations and future prospects

The present study used latent profile analysis to explore the potential categories of college students’ dormitory interpersonal conflict coping styles and the effects of some demographic factors on them, and also to compare the differences in emotional characteristics of different dormitory interpersonal conflict coping groups. This study also has the following limitations: (1) The self-reporting method was used to collect data, which may be affected by the social approval effect. Future research can adopt multi-channel sources to collect data. Furthermore, the study adopted a cross-sectional design, making it difficult to reveal the causal relationships among the variables. Future research can conduct causal inferences by integrating longitudinal research designs and other methods, such as the influence mechanisms between conflict management styles and emotions, and personality traits. (2) The research and investigation scope is limited to a certain university in the northern part of Anhui Province, China. This university is an ordinary, normal undergraduate institution with a relatively high number of female students. Therefore, when the cluster sampling method based on classes is adopted, it naturally leads to a higher number of female students in the research sample. Given the significant gender differences in interpersonal conflict management methods, the classification results of the potential profile analysis in this study and the proportion of people involved may have certain biases. For instance, the proportion of people adopting positive coping styles is higher. Future research should expand the scope of the investigation to include universities of different types and scales. At the same time, pay attention to the balance of the number of men and women. (3) The research results are only applicable to college students in the context of Chinese culture. Chinese society values a collectivist culture and emphasizes the importance of interpersonal harmony, which may have an impact on the interpersonal conflict management methods of Chinese college students. Therefore, the applicability of the research results in other cultural contexts needs to be carefully considered. Future research can conduct cross-cultural studies to compare the potential heterogeneity that may exist in people’s conflict management styles under different cultural backgrounds, such as collectivist culture and individualist culture etc.

## Conclusion

The results of this study suggest that: (1) Most Chinese college students can actively choose positive coping methods to maintain the harmony and stability of dormitory relationships in the face of interpersonal conflicts in dormitories, but some students may still lack effective interpersonal coping strategies, and dormitory interpersonal relationships need to be improved. (2) In the process of coping with dormitory conflicts, the way of cooperation is conducive to the resolution of interpersonal conflicts, which is positively correlated with negative emotions. Competition and avoidance, on the other hand, are not helpful for conflict resolution and are positively correlated with negative emotions. (3) The positive coping group scored significantly lower on negative emotions than the negative coping group.

## Data Availability

The raw data supporting the conclusions of this article will be made available by the authors, without undue reservation.

## Footnotes

^1^ https://www.wjx.cn/
