# Correction: Heterogeneity of dormitory interpersonal conflict coping style and its negative emotional characteristics among college students: based on latent profile analysis

**DOI:** 10.3389/fpsyg.2026.2019508

**Published:** 2026-10-26

**Authors:** Junxing Pan, Xiaoyun Zhao, Cui Lyu, Lili Xu, Ying Zhu

**Affiliations:** 1 Department of Student Affairs, Huaibei Normal University, Huaibei, China; 2 School of Education, Huaibei Normal University, Huaibei, China

**Keywords:** dormitory interpersonal relationships, interpersonal conflict coping style, latent profile analysis, college student, negative emotions

The reference for Bhatra et al. (2023) was erroneously written as Aastha, B. (2023). Significance of compatible room/ flatmate: a qualitative thematic study. *Int. J. Indian Psychol.* 11, 2349–3429. doi: 10.25215/1104.174. It should be:

Bhatra, A., Gupta, A., Bansal, A., and Khan, M. Z. (2023). Significance of compatible room/flatmate: a qualitative thematic study. *Int. J. Indian Psychol.* 11, 1869–1880. doi: 10.25215/1104.174

The reference for Bazezew and Neka (2017) was erroneously written as Bazezew, A., and Neka, M. (2017) Interpersonal conflicts and styles of managing conflicts among students at Bahir Dar University, University of Stellenbosch, South Africa. doi: 10.24085/JSAA.V5I1.2480. It should be:

Bazezew, A., and Neka, M. (2017). Interpersonal conflicts and styles of managing conflicts among students at Bahir Dar University, Ethiopia. *J. Stud. Aff. Afr.* 5, 27–39. doi: 10.24085/JSAA.V5I1.2480

The reference for Hanasono and Nadler (2012) was erroneously written as Hanasono, L. K., and Nadler, L. B. (2007). A dialectical approach to rethinking roommate relationships. *J. Coll. Stud. Dev.* 53, 623–635. doi: 10.1353/csd.2012.0069. It should be:

Hanasono, L. K., and Nadler, L. B. (2012). A dialectical approach to rethinking roommate relationships. *J. Coll. Stud. Dev.* 53, 623–635. doi: 10.1353/csd.2012.0069

The reference for Liao et al. (2012) was erroneously written as Liao, J., Zhou, B., Luo, C., and Yu, Q. (2012). The mediating role of social support in the relationship between personality and interpersonal relationship burnout. *Chin. J. Health Psychol.* 20, 114–116. doi: 10.13342/j.cnki.cjhp.2012.01.033. It should be:

Liao, J., Zhou, B., Luo, C., and Yu, Q. (2012). 大学新生应对方式与宿舍人际困扰的关系研究 [the relationship between college freshmen's coping styles and dormitory interpersonal disturbance]. *Chin. J. Health Psychol*. 20, 114–116. Chinese. doi: 10.13342/j.cnki.cjhp.2012.01.033

The reference for Yang and Zhang (2012) was erroneously written as Yang, S., and Zhang, J. (2012). Relationship between coping styles and cynicism of college students. *Chin. J. Health Psychol*. 20, 1905–1907. doi: 10.13342/j.cnki.cjhp.2012.12.021. It should be:

Yang, S., and Zhang, J. (2012). 大学生宿舍关系与主观幸福感的相关研究 [relationship between dormitory interpersonal relationship and subjective well-being of college students]. *Chin. J. Health Psychol*. 20, 1905–1907. Chinese. doi: 10.13342/j.cnki.cjhp.2012.12.021

The reference for Pan (2009) was erroneously written as Pan, X. (2009). A study on the influence of individual emotion on the tendency of conflict management style—an example of experimental method. *Henan Soc. Sci*. 17, 133–137. doi: 10.13342/j.cnki.cjhp.2017.01.018 It should be:

Pan, X. (2009). 个体情绪对冲突管理方式倾向性的影响研究________以实验法为例 [A study on the influence of individual emotion on the tendency of conflict management style—an example of experimental method]. *Henan Soc. Sci*. 17, 133–137. Chinese.

The reference for Pan and Gao (2017) was erroneously written as Pan, W., and Gao, X. (2017). Relationship among dormitory interpersonal relationship, learning adaptability and academic achievements of undergraduates. *Chin. J. Health Psychol*. 25, 73–76. It should be:

Pan, W., and Gao, X. (2017). 大学生宿舍人际关系、学习适应与学业成就 [relationship among dormitory interpersonal relationship, learning adaptability and academic achievements of undergraduates]. *Chin. J. Health Psychol*. 25, 73–76. Chinese. doi: 10.13342/j.cnki.cjhp.2017.01.018

The reference for The Paper (2024) was erroneously written as Punch News. (2024) Why are Chinese university dormitories stuck in the 1980s? Available online at: https://www.thepaper.cn/newsDetail˙forward˙27211228 (Accessed April 30, 2024). It should be:

The Paper (2024). 中国大学宿舍为啥停留在80年代? [why are Chinese university dormitories stuck in the 1980s?]. Chinese. Available online at: https://www.thepaper.cn/newsDetail˙forward˙27211228 (Accessed April 30, 2024).

The reference for Dong et al. (2012) was erroneously written as Dong, S., Dong, X., Peng, L., Chen, X., and Wang, S. (2012). Interpersonal relationship inside dormitory of college students in Guangzhou. *Chin. J. Sch. Health* 33, 237–238. doi: 10.16835/j.cnki.1000-9817.2012.12.016 It should be:

Dong, S., Dong, X., Peng, L., Chen, X., and Wang, S. (2012). 广州市大学生宿舍人际关系现况分析 [interpersonal relationship inside dormitory of college students in Guangzhou]. *Chin. J. Sch. Health* 33, 1446–1448+1451. Chinese. doi: 10.16835/j.cnki.1000-9817.2012.12.016

The reference for Lei et al. (2018) was erroneously written as Lei, X., Wang, J., Zhang, Y., Ye, B., and Liu, C. (2018). Core self-evaluation and depression: a chain mediating model. *Chin. J. Clin. Psychol*. 4, 808–810. doi: 10.16128/j.cnki.1005-3611.2018.04.039 It should be:

Lei, X., Wang, J., Zhang, Y., Ye, B., and Liu, C. (2018). 核心自我评价对大学生抑郁的影响:应对方式和人际关系困扰的链式中介作用 [core self-evaluation and depression: a chain mediating model]. *Chin. J. Clin. Psychol*. 26, 808–810+830. Chinese. doi: 10.16128/j.cnki.1005-3611.2018.04.039

The reference for Zhang et al. (2012) was erroneously written as Zhang, D., Li, X., and Wei, S. (2012). The relationship between college students' dormitory relationship, learning attitude and learning self-efficacy. *Chin. J. Health Psychol*. 25, 7–76. doi: 10.13342/j.cnki.cjhp.2012.10.023 It should be:

Zhang, D., Li, X., and Wei, S. (2012). 大学生宿舍关系与学习态度和学习效能的关系 [the relationship between college students' dormitory relationship, learning attitude and learning self-efficacy]. *Chin. J. Health Psychol*. 20, 1585–1588. Chinese. doi: 10.13342/j.cnki.cjhp.2012.10.023

The reference for Zhao and Su (2015) was erroneously written as Zhao, Q., and Su, C. (2015). A study of the relationship between competitive attitudes, dormitory interpersonal levels and aggression among college students. *Ludong Univ. J*. (*Philos. Soc. Sci. Ed*.) 11, 31–51. doi: 10.20063/j.cnki.cn37-1452/c.2015.06.008 It should be:

Zhao, Q., and Su, C. (2015). 大学生竞争态度、宿舍人际关系水平和攻击性的关系研究 [a study of the relationship between competitive attitudes, dormitory interpersonal levels and aggression among college students]. *Ludong Univ. J*. (*Philos. Soc. Sci. Ed*.) 32, 31–36+51. Chinese. doi: 10.20063/j.cnki.cn37-1452/c.2015.06.008

The reference for Yang et al. (2024) was erroneously written as Yang, X., Bao, Y., and Wang, D. (2024). Empirical study of the effect of dormitory conflict on dormitory exclusion—the mediation role of competition. *Psychology* 15:14. doi: 10.4236/psych.2024.1511101 It should be:

Yang, X., Bao, Y., and Wang, D. (2024). Empirical study of the effect of dormitory conflict on dormitory exclusion—the mediation role of competition. *Psychology* 15, 1728–1741. doi: 10.4236/psych.2024.1511101

The reference for Yang (2020) was erroneously written as Yang, M. (2020). Relationship between college students interpersonal goals and dormitory interpersonal relationship: mediating role of conflict coping strategies. *Chin. J. Health Psychol*. 28, 1225–1230. doi: 10.13342/i.cnki.cihp.2020.08.025 It should be:

Yang, M. (2020). 大学生人际交往目标与宿舍人际关系的关系:冲突应对方式的中介作用 [relationship between college students interpersonal goals and dormitory interpersonal relationship: mediating role of conflict coping strategies]. *Chin. J. Health Psychol*. 28, 1225–1230. Chinese. doi: 10.13342/j.cnki.cjhp.2020.08.025

The reference for Zhao et al. (2019) was erroneously written as Zhao, D., Wang, T., and Wu, K. (2019). The relation between college students' dormitory interpersonal relationship and depression: the mediating effect of dormitory interpersonal conflict coping style and the moderating effect of gender. *Psychol. Dev. Educ*. 35, 597–604. doi: 10.16187/j.cnki.issn1001-4918.2019.05.11 It should be:

Zhao, D., Wang, T., Wu, K., Li, Z., and Zhou, X. (2019). 大学生宿舍人际关系与抑郁的关系:宿舍冲突应对方式的中介作用和性别的调节作用 [the relation between college students' dormitory interpersonal relationship and depression: the mediating effect of dormitory interpersonal conflict coping style and the moderating effect of gender]. *Psychol. Dev. Educ*. 35, 597–604. Chinese. doi: 10.16187/j.cnki.issn1001-4918.2019.05.11

The reference for Thomas (1976) was erroneously written as Thomas, K. W. (1976). “Conflict and Conflict Management,” in *Handbook of Industrial and Organizational Psychological*, ed. M. Dunnette (Chicago: Rand McNally). It should be:

Thomas, K. W. (1976). “Conflict and conflict management,” in *Handbook of Industrial and Organizational Psychology*, ed. M. Dunnette (Chicago: Rand McNally).

The reference for Golding et al. (2022) was erroneously written as Madeleine, G., Miryha, G. R., and Lisa, G. L. (2022). Negative roommate relationships and the health and wellbeing of undergraduate college students. *J. Public Health Student Capstones*. It should be:

Golding, M., Runnerstrom, M. G., and Ludwig, L. G. (2022). Negative roommate relationships and the health and wellbeing of undergraduate college students. *J. Public Health Stud. Capstones* 1, 20–26.

The reference for Chen et al. (2012) was erroneously written as Chen, W., Hu, H., Guang, Y., and Zeng, X. (2012). Analysis of the characteristics of college students' dormitory conflict management styles. *Chin. J. Sch. Health* 33, 237–238. It should be:

Chen, W., Hu, H., Guang, Y., and Zeng, X. (2012). 大学生宿舍冲突管理方式特点分析 [analysis of the characteristics of college students' dormitory conflict management styles]. *Chin. J. Sch. Health* 33, 237–238. Chinese. doi: 10.16835/j.cnki.1000-9817.2012.02.053

The reference for Wang (2022) was erroneously written as Wang, W. (2022). Investigation and research on current situation of interpersonal conflict among college students in dormitory. *J. Changjiang Inst. Technol*. 39, 70–74. It should be:

Wang, W. (2022). 大学生宿舍人际冲突的现状调查研究 [investigation and research on current situation of interpersonal conflict among college students in dormitory]. J. Changjiang Inst. Technol. 39, 70–74. Chinese. doi: 10.14079/j.cnki.cn42-1745/tv.2022.01.017

The reference for Ding et al. (2024) was erroneously written as Ding, Q., Dong, S., Liu, W., and Tian, J. (2024). Dormitory exclusion and depression in college students: the roles of sense of adequacy and family socioeconomic status. *Psychol. Res*. 17, 182–189. doi: 10.19988/j.cnki.issn.2095-1159.2024.02.010 It should be:

Ding, Q., Dong, S., Liu, Y., and Tian, J. (2024). 宿舍排斥与大学生抑郁 :自我肯定和家庭社会经济地位 [dormitory exclusion and depression in college students: the roles of sense of adequacy and family socioeconomic status]. *Psychological Research* 17, 182–189. Chinese. doi: 10.19988/j.cnki.issn.2095-1159.2024.02.010

The reference for Cen and Nie (2009) was erroneously written as Cen, Y., and Nie, Y. (2009). Relation of college students' cooperation-competition, social-adaptability and career decision-making self-efficacy. *Psychol. Res*. 2, 70–74. It should be:

Cen, Y., and Nie, Y. (2009). 大学生合作竞争倾向与社会适应、职业决策自我效能的关系 [relation of college students' cooperation-competition, social-adaptability and career decision-making self-efficacy]. *Psychological Research* 2, 70–74. Chinese.

The reference for Jia (2009) was erroneously written as Jia, X. (2009). A Study on the Current Situation and Influencing Factors of Interpersonal Conflict Handling in College Students' Dormitories. Xi ‘an: Shaanxi Normal University. It should be:

Jia, X. (2009). 大学生宿舍中人际冲突处理的现状及影响因素研究 [a study on the current situation and influencing factors of interpersonal conflict handling in college students' dormitories]. Xi'an: Shaanxi Normal University. Chinese.

The reference for Fan and Zhang (2003) was erroneously written as Fan, F., and Zhang, X. (2003). Interpersonal conflicts and study on conflict management. *J. China Univ. Mining Technol*. (*Soc. Sci*.) 3, 82–91. It should be:

Fan, F., and Zhang, X. (2003). 人际冲突与冲突管理研究综述 [interpersonal conflicts and study on conflict management]. *J. China Univ. Mining Technol*. (*Soc. Sci*.) 82–91.Chinese.

The reference for Lin et al. (2015) was erroneously written as Lin, Y., Zhao, J., and Tang, X. (2015). A study on the correlation between interpersonal relationships and general self-efficacy in college students' dormitories. *China Adult Educ*. 16, 118–120. It should be:

Lin, Y., Zhao, J., and Tang, X. (2015). 大学生宿舍人际关系与—般自我效能感的相关研究 [a study on the correlation between interpersonal relationships and general self-efficacy in college students' dormitories]. *China Adult Educ*. 118–120. Chinese.

The reference for Wang (2008) was erroneously written as Wang, F. (2008). An empirical research on factors effecting dormitory interpersonal relationship of college student. *Univ. Educ. Sci*. 1, 84–89. It should be:

Wang, F. (2008). 大学生寝室人际关系影响因素的实证研究 [an empirical research on factors effecting dormitory interpersonal relationship of college student]. *Univ. Educ. Sci*. 84–89. Chinese.

The reference for Li and Tang (2010) was erroneously written as Li, L., and Tang, C. (2010). Research of sense of interpersonal conflicts in students' dormitory, process modes and corelations. J. Chengdu Aeronaut. Vocat. Tech. Coll. 26, 11–13. It should be:

Li, L., and Tang, C. (2010). 大学生宿舍人际冲突感、处理方式及其关系的实证研究 [research of sense of interpersonal conflicts in students' dormitory, process modes and corelations]. *J. Chengdu Aeronaut. Vocat. Tech. Coll*. 26, 11–13+56. Chinese.

The original version of this article has been updated.

